# An Innovative Approach to Tissue Processing and Cell Sorting of Fixed Cells for Subsequent Single-Cell RNA Sequencing

**DOI:** 10.3390/ijms231810233

**Published:** 2022-09-06

**Authors:** Ivanina Mutisheva, Steve Robatel, Lukas Bäriswyl, Mirjam Schenk

**Affiliations:** 1 Institute of Pathology, Experimental Pathology, University of Bern, CH-3008 Bern, Switzerland; 2 Graduate School Cellular and Biomedical Sciences, University of Bern, CH-3008 Bern, Switzerland

**Keywords:** single-cell RNA sequencing, fluorescence-activated cell sorting, cell fixation, pancreatic cancer

## Abstract

Although single-cell RNA sequencing (scRNA-seq) is currently the gold standard for the analysis of cell-specific expression profiles, the options for processing, staining, and preserving fresh cells remain very limited. Immediate and correct tissue processing is a critical determinant of scRNA-seq success. One major limitation is the restricted compatibility of fixation approaches, which must not destabilize or alter antibody labeling or RNA content or interfere with cell integrity. An additional limitation is the availability of expensive, high-demand cell-sorting equipment to exclude debris and dead or unwanted cells before proceeding with sample sequencing. The goal of this study was to develop a method that allows cells to be fixed and stored prior to FACS sorting for scRNA-seq without compromising the quality of the results. Finally, the challenge of preserving as many living cells as possible during tissue processing is another crucial issue addressed in this study. Our study focused on pancreatic ductal adenocarcinoma samples, where the number of live cells is rather limited, as in many other tumor tissues. Harsh tissue dissociation methods and sample preparation for analysis can negatively affect cell viability. Using the murine pancreatic cancer model Pan02, we evaluated the semi-automated mechanical/enzymatic digestion of solid tumors by gentleMACS Dissociator and compared it with mechanical dissociation of the same tissue. Moreover, we investigated a type of cell fixation that is successful in preserving cell RNA integrity yet compatible with FACS and subsequent scRNA-sequencing. Our protocol allows tissue to be dissociated and stained in one day and proceeds to cell sorting and scRNA-seq later, which is a great advantage for processing clinical patient material.

## 1. Introduction

Although single-cell RNA sequencing (scRNA-seq) has become very popular for cell profiling of heterogenous cell populations, the development of techniques for storage and delayed processing of fresh samples is an area of active interest with limited current capabilities [1]. In addition to the limited options for cell preservation, methodological limitations affecting sequencing outcomes, such as proper cell processing and sorting, currently persist.

A critical parameter for deep and comprehensive tissue phenotyping through sequencing is the number of available live cells. The viability of isolated cancer tissue samples is relatively low, largely due to local environmental factors. Moreover, various treatments such as neoadjuvant chemo- or radio-therapy have an impact on cell viability [2]. Successful scRNA-seq requires removal of dead cells [3] and debris, so isolation of as many live cells as possible from the initial sample is beneficial [3,4].

Another obstacle to the widespread use of single-cell methodologies, particularly for tumor studies, is the limited availability of assisted fluorescence-activated cell sorting (FACS) machines, which is often difficult to reconcile with surgery schedules. However, FACS for the removal of dead cells and debris or to purify specific cell populations prior to scRNA-seq is often a prerequisite for obtaining high-quality sequencing data [4]. The ability to fix cells prior to subsequent FACS and scRNA-seq greatly increases flexibility in the timing and location of cell sorting and sequencing.

Although several methods are available for preserving fresh tissue and antibody-labeled cells [5], few adequately preserve RNA until further sequencing and are compatible with standard sequencing methods [6]. Inadequate preservation can lead to potentially unfavorable outcomes, such as insufficient cells for sequencing, damaged RNA, or incomplete or incorrect data from the sequencing run. Recently, Attar et al. described a possible solution that permits the preservation of cells for deferred scRNA-seq by fixing them in 3,3-dithio-bis-(sulfosuccinimidyl) propionate (DSP) [7], also known as Lomant’s reagent. This approach has since been validated by several other groups [8,9]. DSP is a reversible chemical crosslinker that is used to fix cells without altering their RNA levels [10]. Later, dithiothreitol (DTT) can be used to cleave the formed disulfide bonds to de-crosslink the amine-containing molecules and revert DSP reaction [11,12]. However, the effect of DSP crosslinking on fluorescent intensity and stability of fluorochrome-conjugated antibody-labeled cells has not yet been described in detail.

In this study, we compared mechanical dissociation with a semi-automated enzymatic/mechanical method of sample digestion using pre-optimized cocktails of enzymes on a gentleMACS^TM^ Dissociator (Miltenyi Biotec, Bergisch Gladbach, Germany). As human pancreatic tissue is very precious, limited, and difficult to obtain, we used fresh murine pancreatic adenocarcinoma (Pan02) tissue to optimize our sample dissociation, staining, preservation, cell sorting, and sequencing protocols. However, the method described in this study for isolation and fixation of primary tumor cells for subsequent scRNA-seq should be applicable to a variety of tissues and cell types, including low cell numbers (e.g., stem cells). We successfully applied it to several samples of human patient material, including primary and metastatic pancreatic cancer, and at low cell numbers. To preserve the cells after dissociation and antibody labeling, prior to FACS sorting and scRNA-seq, we compared DSP-fixed and DSP-fixed DTT-de-crosslinked cells with fresh cells. FACS of DSP-fixed cells was tested independently to determine whether DSP-fixation adversely affected cell sorting. The scRNA-seq of all three samples was performed to compare the sequencing outcomes. The scRNA-seq was performed twice in two independent experiments, and the data are presented both individually and as a merged dataset.

## 2. Results

**Figure 1:**
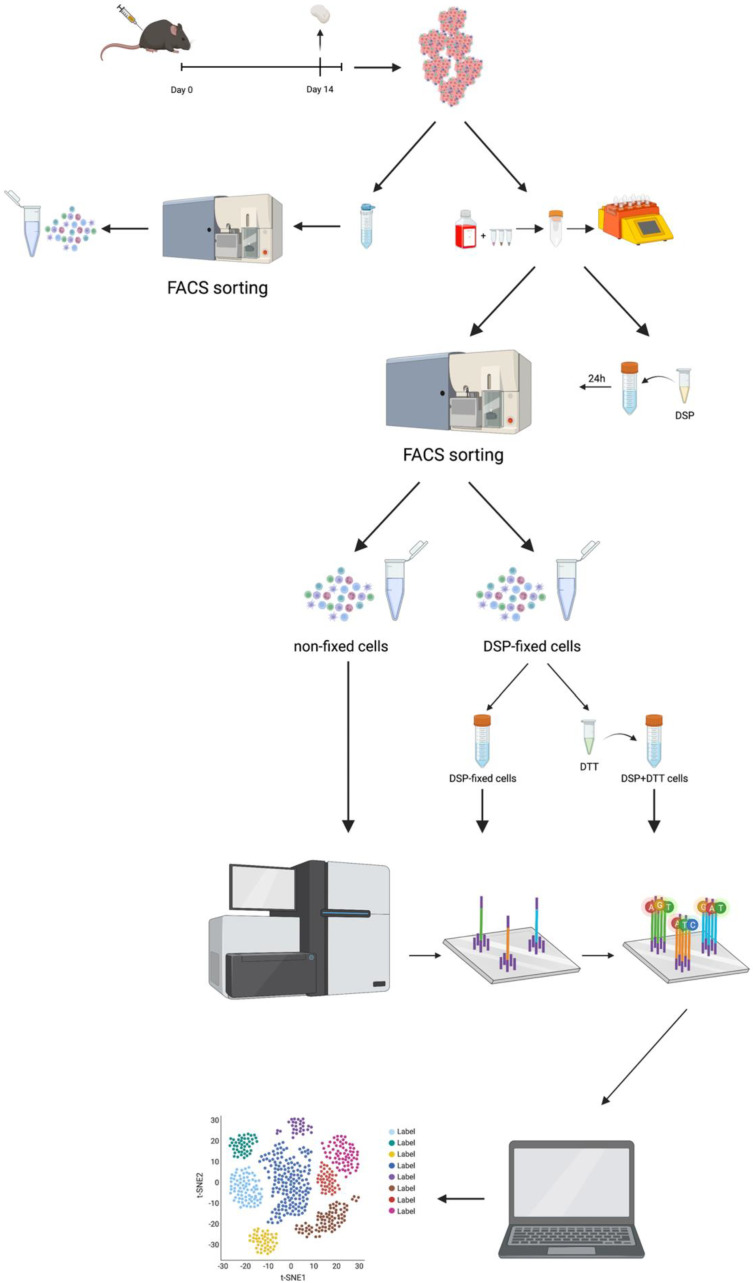
Schematic overview of the tissue processing workflow.

To optimize cell isolation and preservation, we compared different protocols for cell processing and fixation before cell sorting and scRNA-seq analysis. First, the isolation of cells from murine pancreatic tumors (Pan02) by mechanical dissociation was compared with enzymatic/mechanical digestion using a gentleMACS^TM^ Dissociator (Miltenyi Biotec). We also independently investigated whether the DSP fixation process affected FACS or scRNA-seq. Two independent experiments were performed, each with three samples. Figure 1 presents a graphical representation of the workflow.

Tumors were inoculated in mice and harvested on day 14 post-injection. Tumors were either dissociated mechanically or mechanically/enzymatically digested (using gentleMACS^TM^ Dissociator, Miltenyi Biotec) prior to antibody labeling. One sample was DSP fixed and stored for at least 24 h prior to FACS, one sample was directly subjected to FACS for live CD45^+^ immune cells. Half of the DSP fixed samples were de-crosslinked (using DTT) prior to scRNA-seq. All three samples were sequenced on Illumina NovaSeq 6000 sequencer, and data were analyzed using the Seurat package in R.

### 2.1. Tumor Dissociation Protocol Optimization

**Figure 2:**
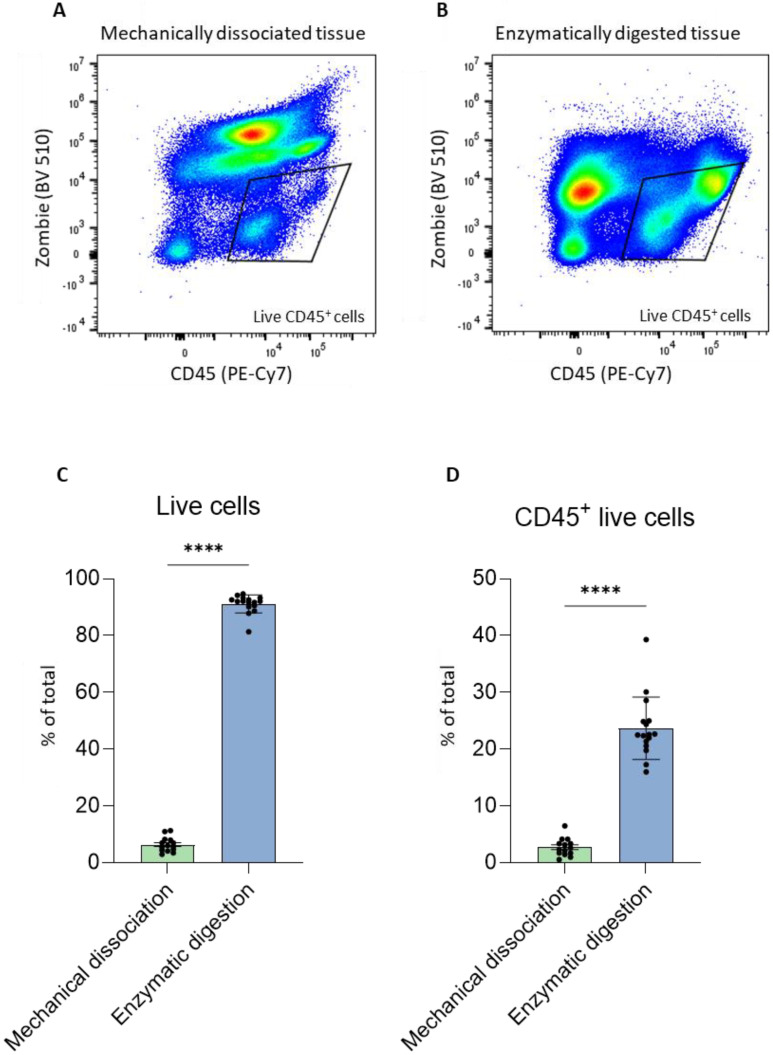
Comparison between mechanical and enzymatic tumor dissociation. FACS gating strategy to assess the percentage of Zombie^−^ (live) and live CD45^+^ cells obtained using mechanical (**A**) and enzymatic/mechanical (using gentleMACS^TM^ Dissociator) (**B**) tumor dissociation. Percentage of Zombie^−^ (live) cells (**C**) and live immune cells (CD45^+^, (**D**)) using mechanical dissociation and enzymatic digestion is shown, each dot represents one tumor. Statistical analysis was performed using Student’s *t*-test; *n* ≥ 15 (per group) from three independent experiments; *p* ≤ 0.0001 (****).

As previously discussed, one of the constraints of studying primary dissociated pancreatic cancer tissue is the limited number of live cells, which we observed to be correlated with the number of live immune cells. Since our goal was to process clinical tissue samples obtained from surgeries for scRNA-seq, it was crucial to first optimize the dissociation and cell recovery steps using a more plentiful tissue source, such as murine Pan02 tumors. Tumors were isolated from the flank of mice on day 14 and divided in half for parallel processing to avoid intertumoral variability and to ensure a relatively similar distribution of cell types in each dissociation method. One half of each tumor was subjected to mechanical dissociation and strained through a filter to obtain a homogeneous cell suspension. The second half of each tumor was enzymatically dissociated using the gentleMACS^TM^ Dissociator according to the manufacturer’s protocol. Cells isolated from each dissociation protocol were subsequently incubated with the dye Zombie Aqua, which enables discrimination between dead and live cells, followed by a fluorochrome-labeled antibody specific for the immune cell marker CD45 to permit sorting of live immune cells from heterogeneous samples. An identical gating strategy to analyze the percentage of live CD45^+^ cells was used for both dissociation protocols (Figure 2A,B). In addition, several tumor cell suspensions from mechanical and mechanical/enzymatic digestion with gentleMACS^TM^ were acquired using FACS and the results were analyzed using FlowJo. Mechanical dissociation resulted in only approximately 10% live cells, whereas mechanical/enzymatic digestion with gentleMACS^TM^ resulted in 90% of the recovered cells being alive (Figure 2C). Moreover, the higher percentage of live cells correlated with an increased percentage of live immune cells (Figure 2D). Therefore, for the remaining experiments, we chose the enzymatic/mechanical method using gentleMACS^TM^ to dissociate tumors, as it resulted in a significantly higher percentage of live cells.

### 2.2. Sorting of DSP-Fixed Cells by FACS

After establishing a dissociation method that vastly improved the recovery of live cells, we next investigated whether DSP crosslinking affected cell viability, FACS staining, or sorting of live immune cells.

Tumor cell suspensions obtained after enzymatic digestion were labeled with Zombie dye and anti-CD45 antibody to distinguish live (zombie-negative) immune cells (CD45^+^) from dead ones. The cell suspensions were divided into two identical samples. The fresh, unfixed cells were immediately subjected to FACS. The other sample was treated with DSP, as previously described [7], stored at 4 °C for at least 24 h, and acquired by FACS the following day (a detailed fixation protocol is provided in the Materials and Methods section).

**Figure 3:**
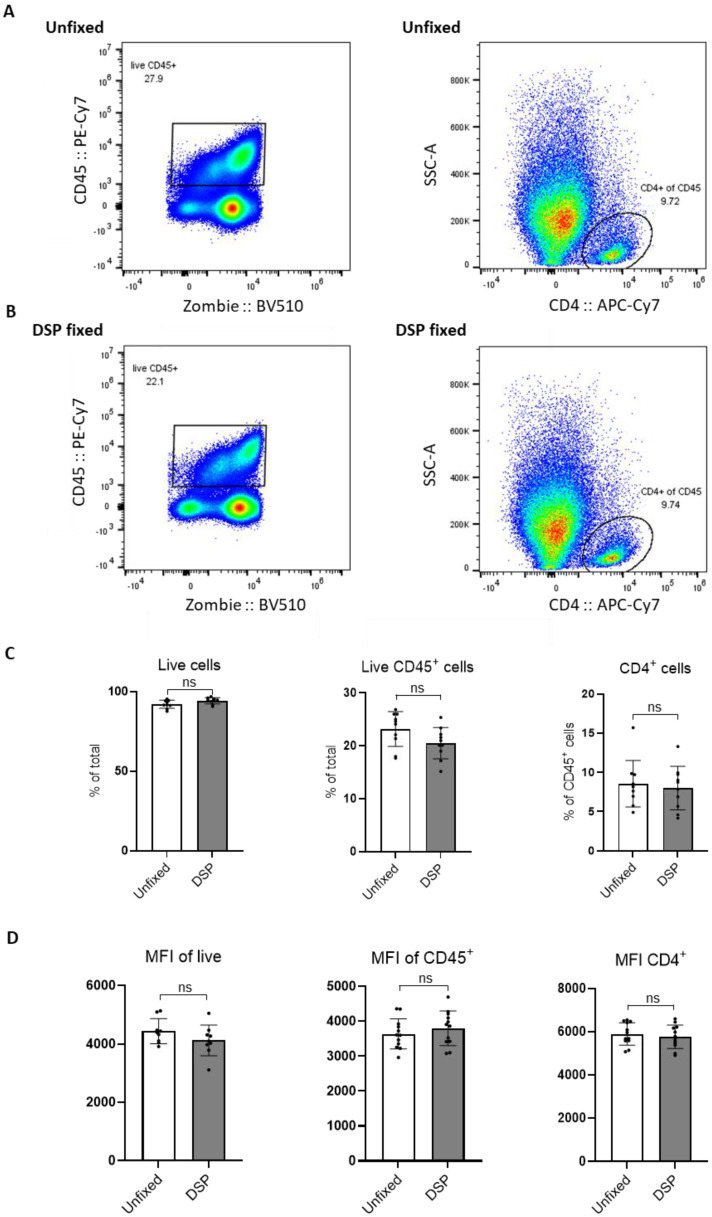
FACS analysis of unfixed and DSP-fixed cells from Pan02 tumors. Pan02 tumors were dissociated and cells processed for FACS. Representative gating strategy for live CD45^+^ and CD4^+^ cells in unfixed (**A**) and DSP-fixed cells. (**B**) Statistical analysis of percentage of live (Zombie^−^), CD45^+^, and CD4^+^ cells (**C**) and MFI of live, CD45^+^, and CD4^+^ in unfixed and DSP-fixed cells (**D**); each dot denotes a separate processed tumor from a mouse; statistical analysis was performed using Student’s *t*-test; *n* = 10 (per group), *p*-value > 0.05 (ns).

FACS analysis of multiple Pan02 tumors gated on live (Zombie^−^), CD45^+^, and CD4^+^ cells in fresh (unfixed, Figure 3A) and DSP-fixed (Figure 3B) samples (for at least 24 h) showed no difference in the percentage of positive cells (Figure 3C). In addition, there was no difference in the MFI of these markers with and without DSP fixation (Figure 3D). Therefore, to proceed with scRNA-seq of fresh and DSP-fixed samples, live immune cells from these two sample groups were sorted using identical settings and gating strategies to avoid bias. Cells from multiple tumors (2–3) were pooled to obtain a sufficient number for scRNA-seq. Cell sorting reports revealed no obvious differences in cellular composition nor staining intensities between unfixed and DSP-fixed cells (Supplemental Figure S1). These data show that DSP fixation did not alter cell morphology (size and granularity), antibody binding, or fluorochrome intensity. Thus, no deleterious effect of DSP crosslinking on FACS was observed after 24 h of fixation. Instead, we found that DSP fixation effectively preserved cell integrity and FACS staining to obtain pure, high-quality samples for subsequent scRNA-sequencing.

### 2.3. QC of Samples before Sequencing

After sorting for live immune cells, all samples were processed for scRNA-seq. Therefore, cells were resuspended according to the 10× Genomics protocol to achieve the maximum targeted cell recovery of 10,000 cells and used at a cell concentration of 1200 cells/µL. An optimal range of cell concentration before sequencing ensures that there are enough cells for sequencing yet prevents cell aggregation (multiplets) which occurs at an undesirably high concentration. Therefore, appropriate resuspension is important for ensuring high-quality sequencing data. 

**Figure 4:**
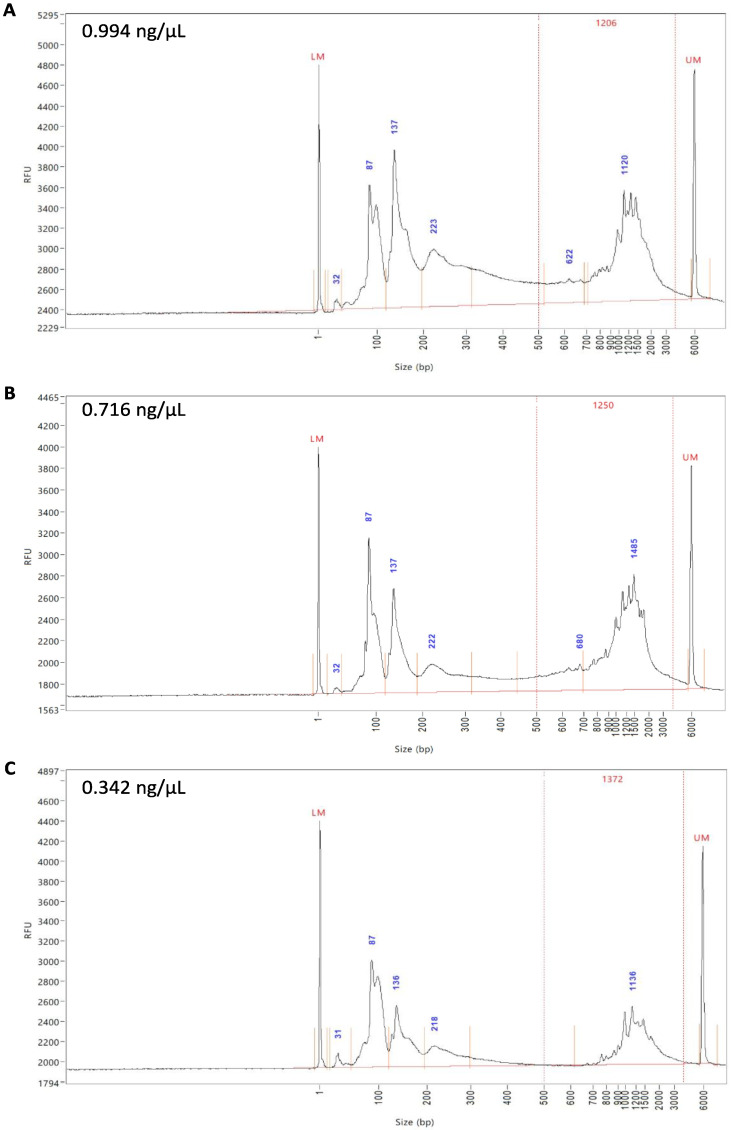
Fragment analysis of the cDNA profiles before library preparation. cDNA profiles of unfixed cells (**A**), DSP-fixed cells (**B**), and DSP-fixed/DTT de-crosslinked cells (**C**) were analyzed for cDNA lengths. cDNA concentration of each processed sample is provided.

To assess whether DTT de-crosslinking affects the subsequent scRNA-seq of samples, half of the DSP-fixed, FACS-sorted samples were de-crosslinked with DTT. All three samples (fresh unfixed, DSP-fixed, and DSP-fixed/DTT de-crosslinked) were subjected to QC after cDNA generation on a Fragment Analyzer^TM^ (Figure 4A–C). As the name suggests, fragment analysis represents the fragments of fluorescently labeled cDNA from the samples separated by automated capillary electrophoresis and then accurately sized by comparison with an internal standard. The expression peaks represent specific cDNA fragments based on their size and expression levels. To demonstrate an intact cDNA library, peaks are to be observed between 400 and 1000 base pairs (bp), which was the case for all three samples (Figure 4A–C). The peaks at ~130 bp were indicative of the presence of empty adaptors, as well as low levels of primer carryover with peaks < 100 bp; therefore, a standard SPRIselect clean-up was performed to remove these primers during library preparation, which improves the signal and allows a better sequencing quality. cDNA quality of the unfixed and DSP-fixed samples was approximately 1 ng/µL (Figure 4A,B) and that of the DSP-fixed/DTT de-crosslinked sample was around 0.34 ng/µL (Figure 4C). These values are all in the optimal range for library preparation and sequencing.

### 2.4. QC of scRNA-Seq Data

**Figure 5:**
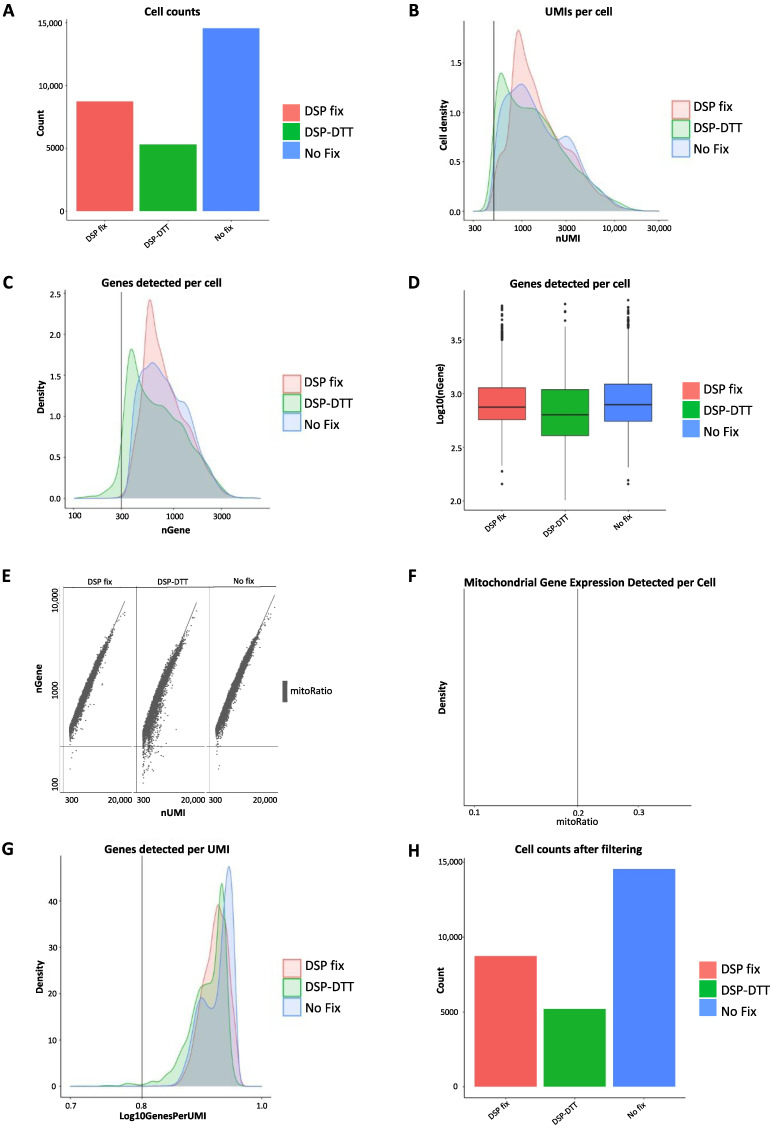
QC of 10× genomics scRNA-seq data. The estimated number of cells in each sample was calculated using the number of unique cellular barcodes detected (**A**). UMIs (transcripts) detected per cell in each sample. The minimum threshold was set at 500 (**B**). Histogram plot of ratio of genes detected per cell density. The minimum cut-off was set to 300 (**C**). Boxplot of average genes detected per cell (**D**). Correlation between genes detected and number of UMIs for each sample (**E**). Visualization of mitochondrial counts detected per cell. Maximum threshold = 0.2 (**F**). Complexity of each test sample, measured in genes detected per UMI with a minimum score > 0.8 (**G**). Filtered cell counts adjusted for all thresholds (**H**).

After confirming that each sample was sufficiently pure and with optimal fragment sizes and peaks, cellular RNA was sequenced using the 10× Genomics scRNA-seq platform on an Illumina sequencer, and the data were subjected to QC (Figure 5). Data were extracted from Cell Ranger (10× Genomics Cell Ranger 3.1.0) and subsequent QC was performed in R using the Seurat package [13]. For the analysis on the pooled datasets, the two experiments were merged using the Seurat package (Supplemental Figure S2). Several metrics were assessed, such as cell counts, the number of unique molecular identifier (UMI) per cell, genes detected per cell, UMIs vs. genes detected, and mitochondrial count ratio based on the Seurat’s recommended default settings. The tests were performed to identify and remove cells of poor quality or insufficient complexity and to obtain only high-quality and biologically relevant cells for further analysis. Data analysis was performed as follows [14]:

First, we determined the cell counts based on the number of unique cellular barcodes recovered. We were able to capture approximately 15,000 cells from the fresh unfixed sample, 10,000 cells from the DSP-fixed sample, and 5000 cells from the DSP-fixed/DTT de-crosslinked sample (Figure 5A). We observed some cell loss, but further controls indicated that RNA quality remained unaffected. 

Second, the number of UMIs per cell was compared. UMIs represent “tags” that are added to DNA fragments upon library preparation and used to identify the input DNA fragment. UMIs are important because they provide error corrections and, therefore, increase the accuracy of sequencing. The lowest threshold was set to 500 UMIs per cell, based on the default recommendations by Seurat, and is indicated by the vertical line at 500 UMIs in the graph (Figure 5B). This threshold indicates the minimum requirement for each sample to be considered for further analysis. The peaks of all three samples exceeded the threshold, and those of the unfixed and DSP-fixed samples largely overlapped, whereas that of the DSP-fixed/DTT de-crosslinked sample was slightly lower. We overlaid the number of genes detected per cell in a histogram (Figure 5C) and visualized them in a boxplot (Figure 5D). The minimal count level was set at 300, and again, we observed an overlap between the unfixed and DSP-fixed samples, and a slightly lower peak in the DSP-fixed/DTT de-crosslinked sample. However, all three samples showed one high peak above 300, which is expected with high-quality data. 

To further assess the quality of the samples, we evaluated the proportion of UMIs among the detected genes. The minimum requirements were set at 300 UMIs and 500 genes (Figure 5E). The cells that did not meet the threshold are displayed in the bottom left quadrant for each sample, indicating poor quality. High-quality cells should contain a high number of UMIs and genes. Almost all the cells met the threshold, and the few cells that did not were excluded from further analysis (Figure 5E). Next, we examined the ratio of mitochondrial gene counts present in any of the cells to assess possible contamination with dying or dead cells that may have passed through cell sorting or died afterwards. The threshold for mitochondrial ratio was set to a maximum possible score of 0.2 (Figure 5F). However, we did not observe any mitochondrial genes in the samples, indicating that cell death or contamination with dead cells was unlikely. The final part of sample QC involves assessing the complexity at the gene level by relating the detected genes to the UMIs. This indicates whether genes are present in many UMIs, which is a requirement for highly complex cells. The minimal threshold value was set at 0.8 to ensure that there would be no contamination with low-complexity cell types (Figure 5G). All three samples passed QC cutoffs, with genes per UMI scores of 0.85 and an overall peak above 0.9, indicating that all samples contained similarly complex populations of RNA, without major outliers. Finally, we filtered the cell counts at the cellular and genetic levels (Figure 5H). We filtered out cells that had <500 UMIs per cell, <300 genes per cell, cells that presented low genes per UMI, and all cells that had a mitochondrial ratio > 0.2. We also removed genes that were expressed in fewer than 10 cells based on our data as they lowered the average complexity of the other cells in which they were not expressed.

Overall, QC demonstrated that the data were of good quality as they met the minimum requirements for passing all QC tests performed. Since all three samples exceeded all minimum QC standards, all samples were considered to have sufficient sequencing quality. Thus, neither DSP fixation nor DSP-DTT de-crosslinking seemed to drastically alter RNA quality, further suggesting that these techniques may be valuable as methods for fixing fresh cells from tissue samples for subsequent cell sorting and scRNA-seq. However, a loss of cells was observed, particularly in the DSP-fixed/DTT de-crosslinked sample, which we address in further detail in the Discussion section. As DTT de-crosslinking did not improve RNA quality and decreased cell number and RNA, it is not reasonable to include this extra step in the protocol, which requires additional time and effort.

### 2.5. Analysis of scRNA-Seq Data

**Figure 6:**
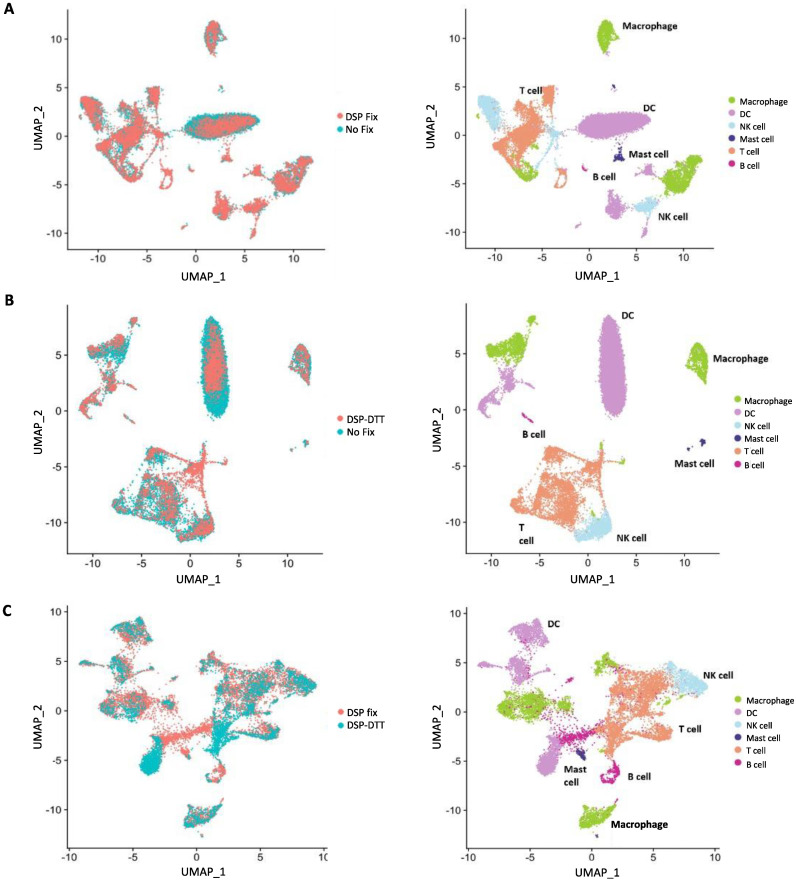
Uniform manifold approximation and projections. Representation (from left to right) of an overlay of cell dispersion, clustering, and cell type annotation for the analyzed samples: unfixed cells compared to DSP-fixed cells (**A**), unfixed cells compared to DSP-fixed/DTT de-crosslinked cells (**B**), and DSP-fixed cells compared to DSP-fixed/DTT de-crosslinked cells (**C**).

QC of the scRNA-seq data showed that all samples were suitable for further downstream analysis. Next, we investigated whether the fixation protocol caused qualitative, i.e., differences in biological analysis between samples. A preliminary analysis was performed using the Seurat package in R [13]. We aimed to discriminate any variance that might have arisen in the dispersion or clustering of cells, which were represented by uniform manifold approximation and projections (UMAP). We compared both the DSP-crosslinked cells and the DSP-DTT treated cells to unfixed cells and the DSP-fixed sample to the DSP-fixed/DTT de-crosslinked sample (Figure 6A–C).

First, in the analysis of cell dispersion, we found a considerable similarity in the superposition obtained under all conditions investigated in both independent experiments (Figure 6 and Supplemental Figure S3). The greatest degree of overlap was exhibited by the DSP-fixed cells and the non-fixed cells (Figure 6A and Supplemental Figure S3A). Nonetheless, the overlay in the other two groups was also high (Figure 6B,C and Supplemental Figure S3B,C). The observed divergence in overlap may be due to differences in cell numbers in the DSP-DTT-treated group, as the DSP-fixed and non-fixed groups showed the highest cell numbers. Clustering analysis of the cells also showed similarity in all three groups, with very similar proportions of cell types and few differences (Figure 6A–C and Supplemental Figure S3A–C). Cell dispersion and clustering analysis of the merged datasets also showed a significant similarity in the superposition and the clustering (Supplemental Figure S4A,B). In addition, proportion analysis, which indicates the percentage of cells in each sample (merged data) assigned to each cluster, showed no significant differences between the unfixed and DSP-fixed samples (Supplemental Figure S4C,D). The only significant changes were observed between the DSP fixed and the DSP-fixed/DTT de-crosslinked samples for the Mast cell and DC cluster (Supplemental Figure S4C,D). Together, these results indicate that DSP fixation does not significantly alter cell viability, clustering, or dispersion of cells. DSP crosslinking was visibly more similar to unfixed cells than to DSP-fixed/DTT de-crosslinked cells in terms of cell clustering. We also performed pseudo-bulk and cluster-specific differential gene expression analysis to assess if either DSP fixation or DSP-DTT treatment affected sequencing results of the samples. Comparison between the three groups revealed a minimal number of differentially expressed genes (DEGs) in pseudo-bulk (Supplemental Figure S5A) and for all cell clusters (Supplemental Figure S5B and Table S1). 

In summary, we concluded that DSP fixation does not affect cell sorting, RNA quality, and sequencing results and DTT de-crosslinking does not provide additional benefits.

## 3. Discussion

The goal of this study was to establish a protocol for conserving primary tissue cells for deferred cell sorting and scRNA-seq analysis. An essential consideration was that the experimental manipulations for storage should not compromise the quality or fidelity of the resulting data. Such a method would permit more flexible sorting and sequencing of precious clinical tissue material to circumvent day-to-day obstacles, such as the availability of necessary equipment. In addition, we optimized several parameters, including dissociation protocol and sorting parameters, and found that tissue dissociation by semi-automated mechanical/enzymatic digestion provided superior yields while generally preserving cell viability. Moreover, we discovered that crosslinking cells using DSP preserved RNA integrity and was compatible with FACS (including the removal of dead cells) prior to sequencing. Although DSP crosslinking can be reversed by adding DTT, in our experiments, this treatment did not improve the sample quality and instead resulted in a loss of cells. Although not described in the current paper, this method of isolating and fixing primary tumor cells for subsequent scRNA-seq should be applicable to a variety of tissues and cell types.

As demonstrated in this study, optimization of the tissue dissociation protocol can dramatically improve the yield of viable cells from each sample. The use of Miltenyi Biotec’s gentleMACS^TM^ Dissociator offers a tremendous advantage over mechanical dissociation alone, resulting in an almost nine-fold increase in the number of live cells. Cell viability is of critical importance because clinical samples from patients are generally limited. Cancer tissues present additional challenges of having a low number of viable cells and few immune cells. Therefore, the ability to dissociate a sample with the least possible loss of viable cells is critical for further analysis. An additional benefit of the gentleMACS^TM^ Dissociator is that its semi-automation can reduce variability in yields and handling between samples or between different users. 

The lack of a suitable method to process a tissue sample and preserve cellular and RNA integrity for subsequent scRNA-seq is a major bottleneck, especially as scRNA-seq has become a preferred method of analysis [15]. Here, we optimized several parameters of a method for cell fixation using DSP previously reported by Attar et al. using unsorted K562 cells, an immortalized leukemia cell line, and sequencing on a different sequencing platform (Fluidigm C1 system) [7]. Here, we showed that primary cells from mouse Pan02 tumors can be successfully crosslinked using DSP and stored prior to FACS and scRNA-seq. DSP crosslinking did not appear to compromise the integrity of cellular RNA from our primary tumor sample, and the sequencing results were comparable to those obtained from unfixed samples. We further tested whether de-crosslinking DSP-fixed cells using DTT could further improve the scRNA-seq results. However, de-crosslinking led to a lower cell recovery and lower cDNA concentrations than DSP-fixation alone. Quality control revealed that all the samples were of high quality. Overall, our data suggest that de-crosslinking the DSP-fixed samples is not required, and additional handling may contribute to cell loss. 

We utilized an additional step of cell labeling and sorting prior to scRNA-sequencing to further refine sample purity. FACS was implemented to remove undesired cells and debris from the sample [16,17,18] and to sort live CD45^+^ cells from our tumor cell suspension. The utility of this step underscores the need for a fixation method that not only preserves the cells and their RNA for subsequent sequencing, but is also suitable for sorting, i.e., it does not interfere with the binding of cell-surface-directed antibodies or fluorochromes. The method described in this study will be of use precisely because of the urgent and timely requirement for a FACS sorter, sequencing device, and specialists for processing surgical samples. The parameters we defined here, namely dissociation using mechanical/enzymatic processing and fixation with DSP (without de-crosslinking) prior to cell sorting (live CD45^+^ cells) and scRNA-seq on an Illumina NovaSeq 6000 sequencer, represent a progressive method.

In conclusion, the approach detailed in this study should allow the much-needed flexibility in planning and execution of scRNA-seq experiments with human surgical samples and other samples with complex scheduling by maintaining cell and RNA integrity during sample storage to permit high-quality sequencing with excellent results at later time points.

## 4. Materials and Methods

### 4.1. Tissue Culture

Murine Pan02 pancreatic cancer cells (a gift from Dr. Ivo Partecke, University of Greifswald, Germany) were cultured in complete RPMI-1640 medium (Sigma-Aldrich, Buchs, Switzerland) supplemented with 10% FBS, 100 units/mL penicillin, 100 μg/mL streptomycin, 1 mM sodium pyruvate, and 2 mM L-glutamine.

### 4.2. Mice, Tumor Inoculation, and Isolation

C57BL/6 mice were purchased from Janvier Labs (Saint Berthevin Cedex, France). The 8–12-week-old age- and sex-matched animals were used for all experiments. On day 0, tumors were engrafted by subcutaneous injection of 2 × 10^5^ Pan02 cells into the left flank of mice. Mice were euthanized on day 14 post-tumor inoculation, and the tumors were isolated and processed. Tumors were either mechanically dissociated by cutting them into small pieces and filtered through a 40 µM strainer (Thermo Fisher Scientific) using a soft rubber pestle and PBS (mechanical dissociation). For the mechanical/enzymatic digestion using the gentleMACS^TM^ Dissociator and tumor Dissociation kit (Miltenyi), the enzyme mix was prepared by adding 2.35 mL of RPMI 1640, 100 µL of Enzyme D, 50 µL of Enzyme R and 12.5 µL of Enzyme A into a gentleMACS C tube. Fat, fibrous, and necrotic areas were removed from the tumor sample and tumors were cut into 2–4 mm pieces and placed into a gentleMACS C tube (Miltenyi) containing the enzyme mix. The C Tube was attached upside down onto the sleeve of the gentleMACS Dissociator. Tumors were dissociated using the program ‘Though 37C_m_TDK_2’ on the gentleMACS^TM^ Octo Dissociator with Heaters. Cells were filtered through a 70 µm cell strainer to discard any remaining cell clumps or tissue before proceeding with the FACS protocol. All mice were housed under specific pathogen-free conditions in the Central Animal Facility of the University of Bern, Switzerland. All animal experiments were performed in accordance with federal regulations and were approved by the Cantonal Veterinary Office (BE70/19).

### 4.3. Flow Cytometric Analyses and Cell Sorting

Tumors were established and harvested as previously described and dissociated either mechanically or mechanical/enzymatic digestion (gentleMACS^TM^ Dissociator) before being filtered through a 40μM strainer (Thermo Fisher Scientific, Waltham, MA, USA). Zombie Aqua or UV Fixable Viability dye (Zombie dye, BioLegend, San Diego, CA, USA) was used to discriminate dead cells (Zombie positive). Anti-mouse CD45.2 monoclonal antibody (clone 104, 109830, BioLegend, San Diego, CA, USA, dilution 1:100, host: mouse,) was used to label immune cells. Samples were acquired using a CytoFLEX S flow cytometer (Beckman Coulter, Brea, CA, USA) and analyzed using FlowJo (Tree Star, version 10.5.2, BD Life Sciences, Ashland, Oregon, USA). FACS before scRNA-seq was performed using a Beckman Coulter MoFlo ASTRIOS BSL-2 cell sorter at the FACS Lab, Department of BioMedical Research (DBMR), University of Bern, Bern, Switzerland.

### 4.4. DSP-Crosslinking and DTT De-Crosslinking

For crosslinking of the experimental samples, the harvested and processed tumor cell suspensions were subjected to a fixation protocol using DSP (Thermo Fisher Scientific, 22585). DSP de-crosslinking was achieved using DTT (Sigma Aldrich, Buchs, Switzerland, D9779), as described in detail by Attar et al. [7]. Briefly, a DSP stock solution (50×) was prepared in 100% anhydrous DMSO (50 mg/mL, stored at −80 °C). Immediately before use, the 50× DSP stock solution was diluted with PBS to working concentration (1 mg/mL) by adding 490 μL PBS to 10 μL DSP (50× stock) dropwise using a 200 μL pipette while vortexing, ensuring minimal precipitation. In the case of substantial precipitation, the dilution was started over with a new DSP stock aliquot. The 1× DSP solution was filtered using a 30 μm filter (Pre-Separation Filters, Miltenyi Biotec) and placed on ice. A total of 200,000 cells were dispensed into a 1.5 mL Eppendorf tube. The cells were then pelleted and washed twice with PBS. The cell pellet was gently resuspended in 200 µL 1× DSP and incubated at 20–22 °C for 30 min. To quench the crosslinker, 4.1 µL of 1 M Tris HCl, pH 7.5 (final concentration 20 mM) was added and gently mixed by pipetting. The fixed cells were stored at 4 °C until further processing. For de-crosslinking, DTT was added to a final concentration of 50 mM and incubated at 37 °C for 30 min.

### 4.5. The 10× Genomics Illumina scRNA-Seq

Samples for scRNA-seq were submitted to the NGS platform at the University of Bern. They performed Gel Bead-in-Emulsion generation, quality control (QC), barcoding of the cells (performed on a Chromium Controller, Single Cell 3′ v3), and finally the sequencing on an Illumina NovaSeq 6000 sequencer, according to the 10× manufacturer protocol.

### 4.6. scRNA-Seq Data Analysis

Raw reads were aligned using a pre-built reference for mouse genomes (refdata-gex-mm10-2020-A; Mouse reference, mm10 (GENCODE vM23/Ensembl 98)) using CellRanger (version 6.0.1, 10× Genomics Inc., Pleasanton, CA, USA). All downstream analyses, including QC were performed using the Seurat package (version 4.1.1) [13] in R (version 4.1.0) as previously described [19]. For cluster annotation, differentially expressed cluster marker genes were extracted using the Seurat package and applied to the gene set enrichment analysis tool Enrichr [20,21].

## 5. Conclusions

In conclusion, the approach detailed in this study should allow much-needed flexibility in planning and executing scRNA-seq experiments with human surgical samples and other samples with complex schedules by maintaining cellular and RNA integrity during sample storage to permit high-quality sequencing at later time points.

## Data Availability

The data presented in this study are available on request from the corresponding author.

## Supplementary Materials

The following supporting information can be downloaded at: https://www.mdpi.com/article/10.3390/ijms231810233/s1.

## Abbreviations

MFI—mean fluorescent intensity; scRNA-seq—single-cell RNA sequencing; FACS—fluorescence-activated cell sorting; DSP—3,3-dithio-bis-(sulfosuccinimidyl)propionate; DTT—dithiothreitol; QC—quality control; NGS—next-generation sequencing; UMI—unique molecular identifier.

## References

[1] Florell S.R., Coffin C.M., Holden J.A., Zimmermann J.W., Gerwels J.W., Summers B.K., Jones D.A., Leachman S.A. (2001). Preservation of RNA for Functional Genomic Studies: A Multidisciplinary Tumor Bank Protocol. Mod. Pathol..

[2] Robatel S., Schenk M. (2022). Current Limitations and Novel Perspectives in Pancreatic Cancer Treatment. Cancers.

[3] Haque A., Engel J., Teichmann S.A., Lönnberg T. (2017). A practical guide to single-cell RNA-sequencing for biomedical research and clinical applications. Genome Med..

[4] 10× Genomics, “What Are the Best Practices for Flow Sorting Cells for 10× Genomics Assays?”. https://kb.10xgenomics.com/hc/en-us/articles/360048826911-What-are-the-best-practices-for-flow-sorting-cells-for-10x-Genomics-assays-.

[5] Yale School of Medicine “Tissue Processing Methods,” Pathology. https://medicine.yale.edu/pathology/ypts/tpd/methods/.

[6] Xiang C.C., Mezey E., Chen M., Key S., Ma L., Brownstein M.J. (2004). Using DSP, a reversible cross-linker, to fix tissue sections for immunostaining, microdissection and expression profiling. Nucleic Acids Res..

[7] Attar M., Sharma E., Li S., Bryer C., Cubitt L., Broxholme J., Lockstone H., Kinchen J., Simmons A., Piazza P. (2018). A practical solution for preserving single cells for RNA sequencing. Sci. Rep..

[8] Gerlach J.P., van Buggenum J.A.G., Tanis S.E.J., Hogeweg M., Heuts B.M.H., Muraro M.J., Elze L., Rivello F., Rakszewska A., van Oudenaarden A. (2019). Combined quantification of intracellular (phospho-)proteins and transcriptomics from fixed single cells. Sci. Rep..

[9] Nesterenko P.A., McLaughlin J., Cheng D., Bangayan N.J., Sojo G.B., Seet C.S., Qin Y., Mao Z., Obusan M.B., Phillips J.W. (2021). Droplet-based mRNA sequencing of fixed and permeabilized cells by CLInt-seq allows for antigen-specific TCR cloning. Proc. Natl. Acad. Sci. USA.

[10] Mattson G., Conklin E., Desai S., Nielander G., Savage M.D., Morgensen S. (1993). A practical approach to crosslinking. Mol. Biol. Rep..

[11] Cleland W.W. (1964). Dithiothreitol, a New Protective Reagent for SH Groups. Biochemistry.

[12] Alliegro M.C. (2000). Effects of Dithiothreitol on Protein Activity Unrelated to Thiol–Disulfide Exchange: For Consideration in the Analysis of Protein Function with Cleland’s Reagent. Anal. Biochem..

[13] Hao Y., Hao S., Andersen-Nissen E., Mauck W.M., Zheng S., Butler A., Lee M.J., Wilk A.J., Darby C., Zager M. (2021). Integrated analysis of multimodal single-cell data. Cell.

[14] 10× Genomics, “Technical Note—Resolving Cell Types as a Function of Read Depth and Cell Number”. https://www.10xgenomics.com/support/single-cell-gene-expression/documentation/steps/sequencing/resolving-cell-types-as-a-function-of-read-depth-and-cell-number.

[15] Kharchenko P.V. (2021). The triumphs and limitations of computational methods for scRNA-seq. Nat. Methods.

[16] Wang A., Middlebrook A., Pennebaker K., Chang C., Wang A., Middlebrook A., Pennebaker K., Chang C., Shum E., Fan C. A Complete Workflow from Single Cell Isolation to mRNA Sequencing Analysis. 2016 [White Paper]. http://cdn.technologynetworks.com/tn/Resources/pdf/a-complete-workflow-from-single-cell-isolation-to-mrna-sequencing-analysis.pdf.

[17] BD Genomics (2021). BD® Single-Cell Multiomics Bioinformatics Handbook.

[18] Kannan S., Miyamoto M., Lin B.L., Zhu R., Murphy S., Kass D.A., Andersen P., Kwon C. (2019). Large Particle Fluorescence-Activated Cell Sorting Enables High-Quality Single-Cell RNA Sequencing and Functional Analysis of Adult Cardiomyocytes. Circ. Res..

[19] Butler A., Hoffman P., Smibert P., Papalexi E., Satija R. (2018). Integrating single-cell transcriptomic data across different conditions, technologies, and species. Nat. Biotechnol..

[20] Chen E.Y., Tan C.M., Kou Y., Duan Q., Wang Z., Meirelles G.V., Clark N.R., Ma’Ayan A. (2013). Enrichr: Interactive and collaborative HTML5 gene list enrichment analysis tool. BMC Bioinform..

[21] Kuleshov M.V., Jones M.R., Rouillard A.D., Fernandez N.F., Duan Q., Wang Z., Koplev S., Jenkins S.L., Jagodnik K.M., Lachmann A. (2016). Enrichr: A comprehensive gene set enrichment analysis web server 2016 update. Nucleic Acids Res..

