# Supplementary material for: An Innovative Approach to Tissue Processing and Cell Sorting of Fixed Cells for Subsequent Single-Cell RNA Sequencing
Source: Int J Mol Sci. 2022 Sep 6;23(18):10233. doi: 10.3390/ijms231810233 (PMC9499188; doi:10.3390/ijms231810233)
Supplement: Supplementary file 1 [file ijms-23-10233-s001.zip › ijms-1830625-supplementary.pdf]

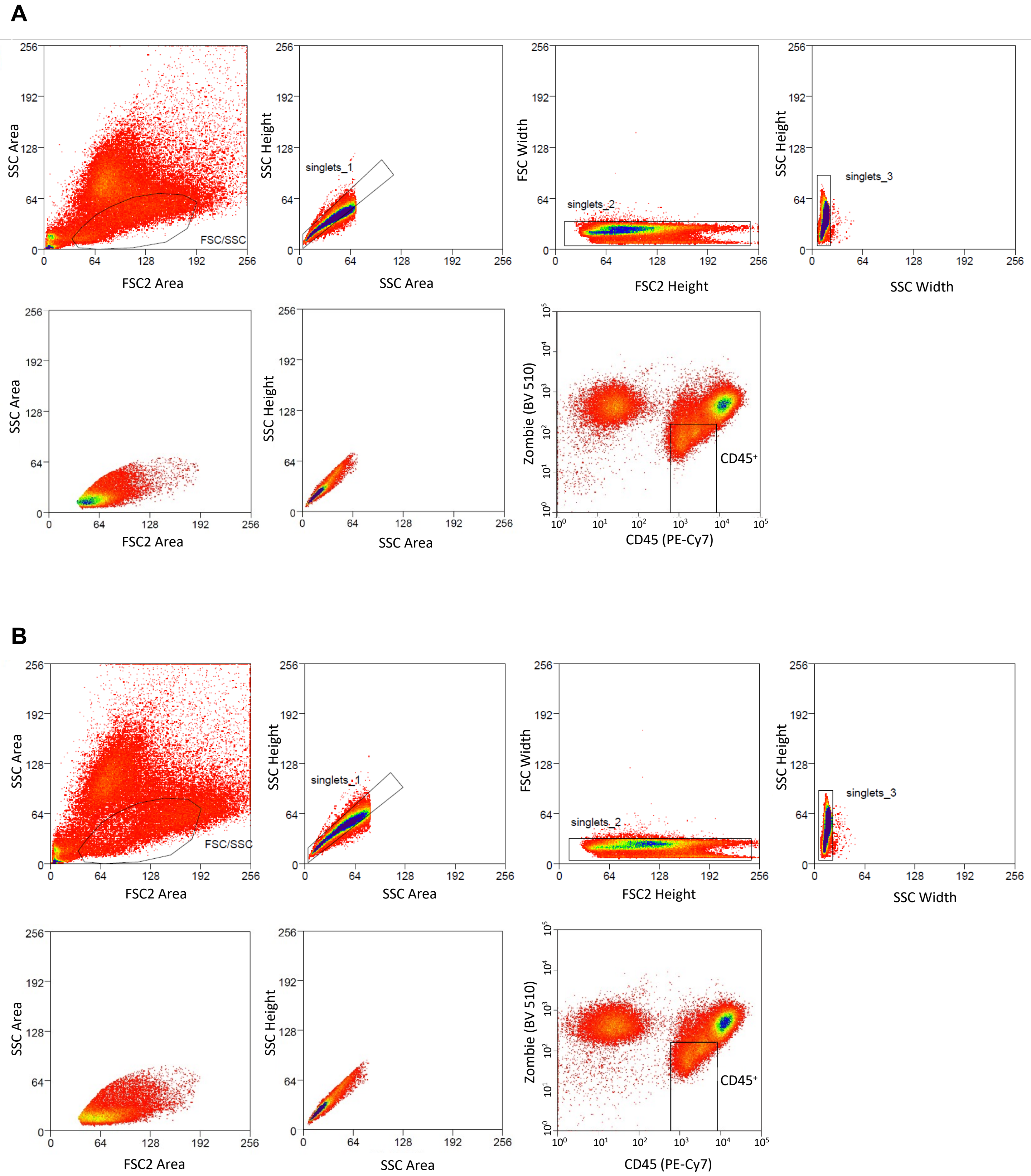

Supplemental Figure S1: **Gating strategy for sorting live CD45$^+$ cells from whole tissue samples**
Summary of gating strategy for sorting live CD45$^+$ cells from fresh unfixed (A) and DSP-fixed samples (B). Forward scatter (FSC) and side scatter (SSC) gates were set to include immune cells. Gating on single cells was performed by SSC-H vs. SSC-A, FSC-H vs. FSC-W, and SSC-W vs. SSC-H gating. The gate for sorting was set to CD45-PE-Cy7$^+$ and Zombie– cells. The cell sorting and scRNA-seq experiments were run in two independent experiments, data from one representative experiment are shown.

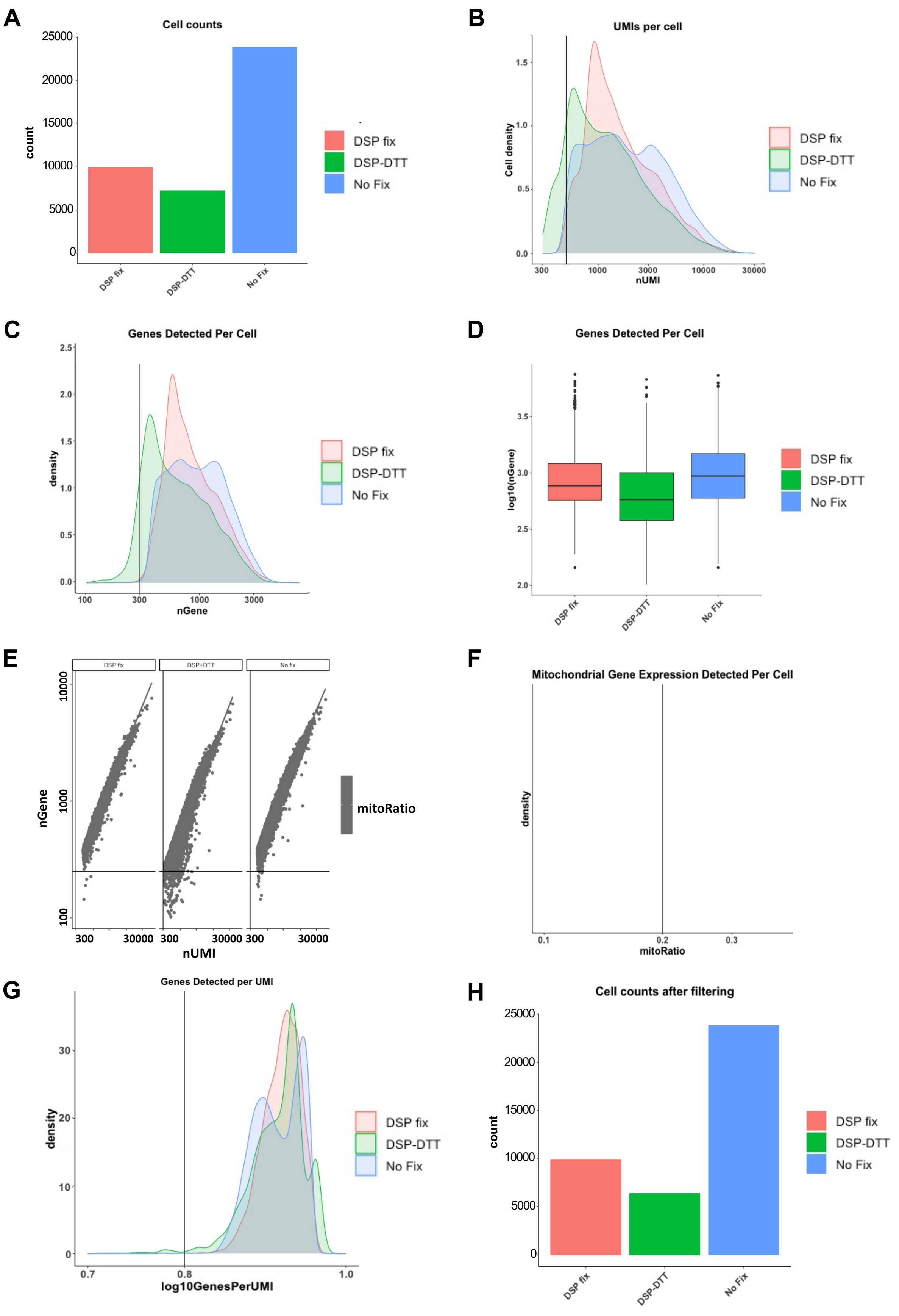

Supplemental Figure S2: QC of 10x Genomics scRNA-seq data of merged datasets
The estimated number of cells in each condition from two independent experiments was calculated by the number of unique cellular barcodes detected (A). UMIs (transcripts) detected per cell in each sample. Minimum threshold was set at 500 (B). Histogram plot of ratio of genes detected per cell density. Minimum cut-off was set to 300 (C). Boxplot of average genes detected per cell (D). Correlation between genes detected and number of UMIs for each sample (E). Visualisation of mitochondrial counts detected per cell. Maximum threshold= 0.2 (F). Complexity of each test sample, measured in genes detected per UMI with a minimum score >0.8 (G). Filtered cell counts adjusted for all thresholds (H).

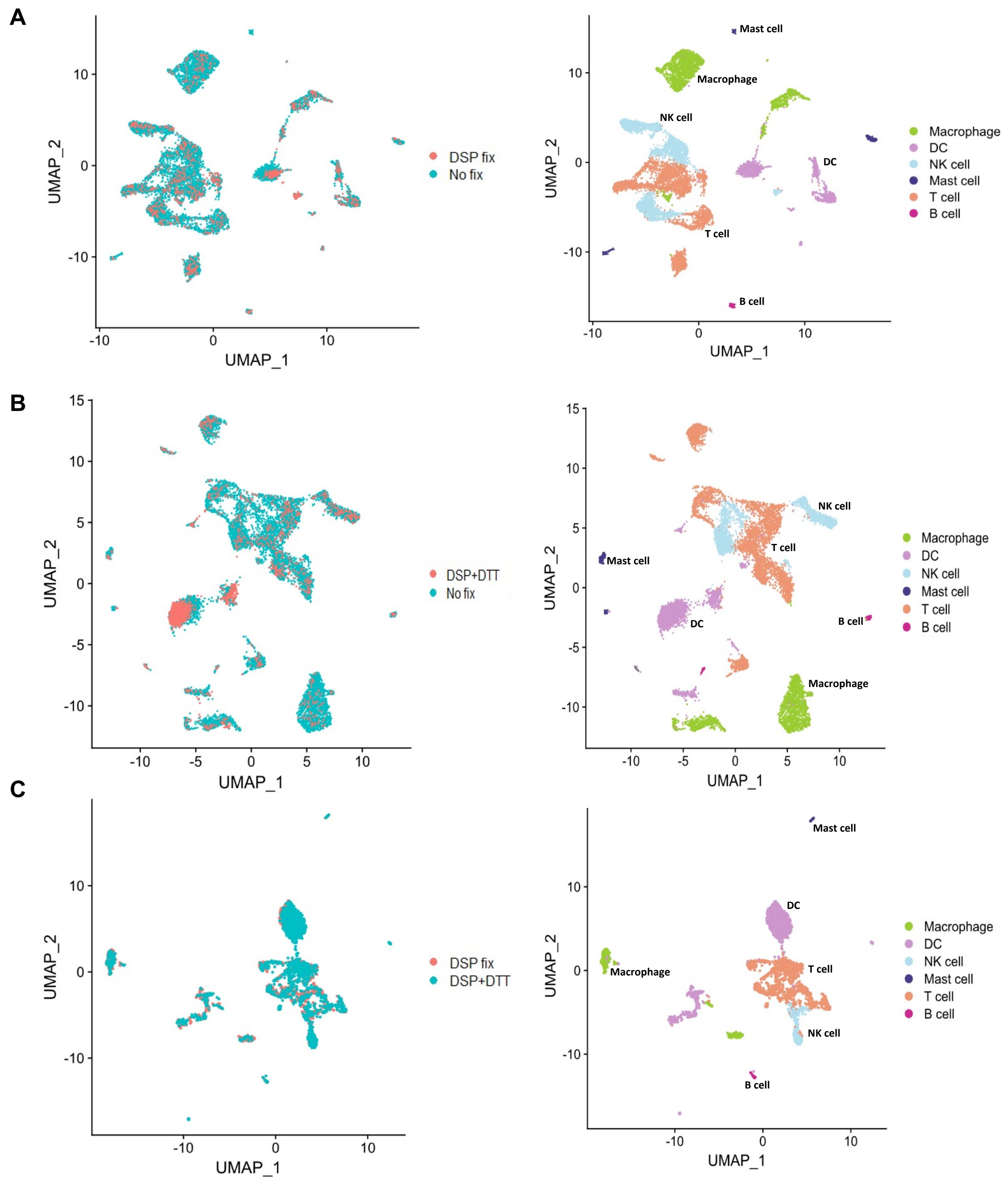

Supplemental Figure S3. Uniform manifold approximation and projections (biological replicate)
Representation (from left to right) of an overlay of cell dispersion, clustering, and cell type annotation for the analyzed samples: unfixed cells compared to DSP-fixed cells (A), un-fixed cells compared to DSP-fixed/DTT de-crosslinked cells (B), and DSP-fixed cells compared to DSP-fixed/DTT de-crosslinked cells (C).

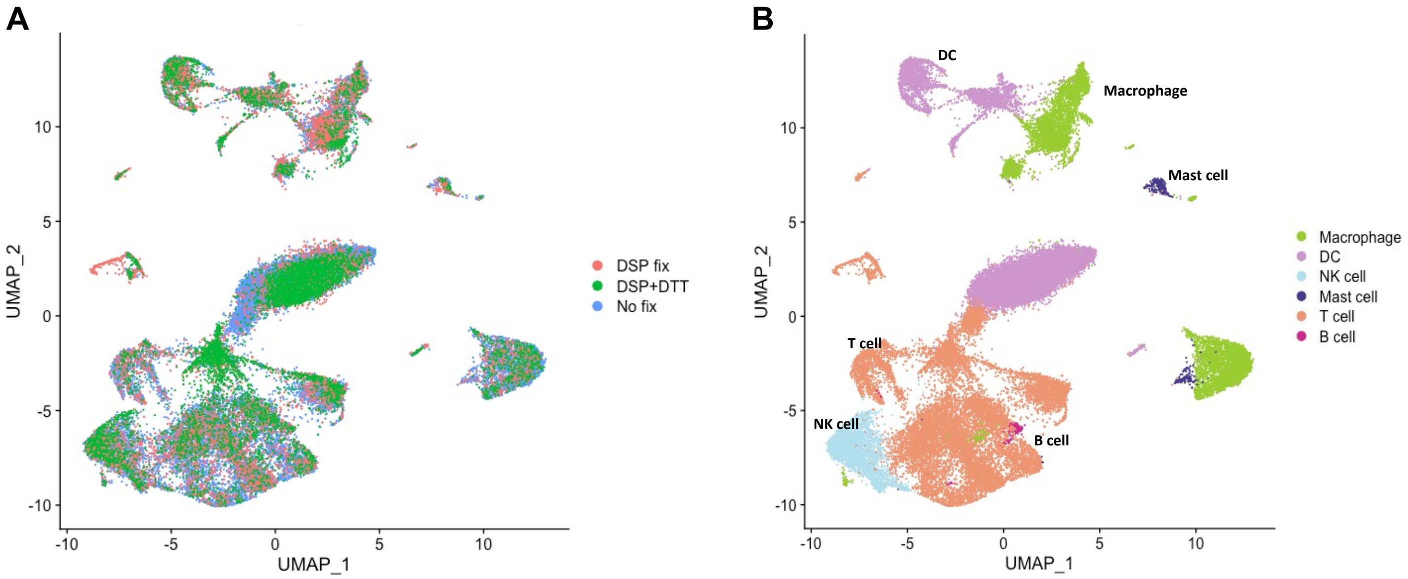

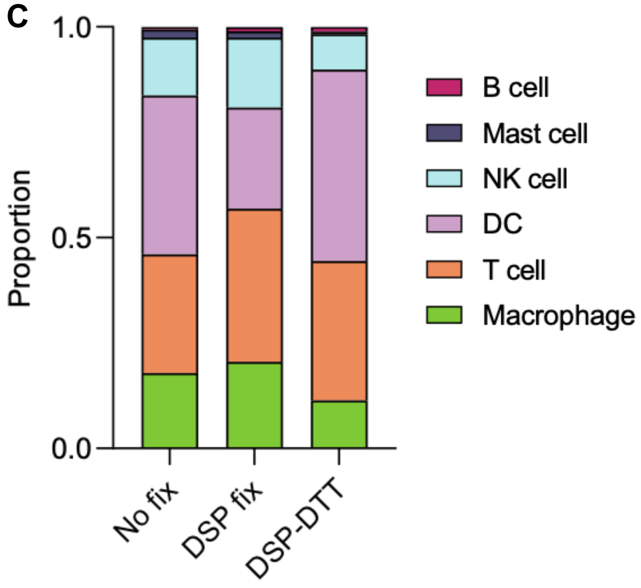

D

P- values			
Sample / Cell cluster	No Fix vs. DSP	No Fix vs. DSP-DTT	DSP vs. DSP-DTT
B cell	0.3442	0.1845	0.3913
DC	0.3186	0.5840	0.0168
Macrophage	0.5846	0.1311	0.0725
Mast cell	0.5458	0.0868	0.0037
NK cell	0.7069	0.2777	0.1406
T cell	0.3646	0.4870	0.6928

Supplemental Figure S4. Uniform manifold approximation and projections and cell proportion analysis of merged datasets
Plot of an overlay of cell dispersion, clustering (A), and cell type annotation (B) for unfixed cells, DSP-fixed cells, and DSP-fixed/DTT de-crosslinked cells from each of two independent merged experiments. Proportion plots indicating the percentage of cells in each sample assigned to each cluster (C). Table showing *p*-values of statistical analysis of differences between in the cell clusters between the groups as assessed by Student's t-test (n=2).

A

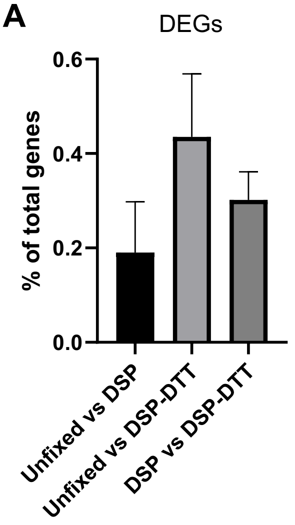

B

**% DEGs of total**			
Sample / Cell cluster	No Fix	DSP	DSP-DTT
B cell	0	0	0
DC	0.090	0.040	0.053
Macrophage	0.167	0.059	0.046
Mast cell	0	0	0
NK cell	0.223	0.012	0.028
T cell	0.195	0.214	0.056

Supplemental Figure S5. Pseudo-bulk and cluster-specific differential gene expression analysis Percentage of differentially expressed genes (DEGs) of all annotated genes between unfixed, DSP-fixed, or DSP-DTT treated samples obtained by pseudo-bulk analysis (A), and for each cluster (B); No DEGs were detected in the B cell and mast cell cluster between the groups; filtered by adjusted p-value < 0.05, n=2.

Supplemental Table S1. DEGs of all annotated genes between different groups

DEGs Macrophage cluster

gene	p_val	avg_log2FC	p_val_adj	sample
Ighg2c	0	0.858	0	No fix
Cyp11a1	0	0.836	0	No fix
Gzmf	0	0.806	0	No fix
Hist1h1b	0	0.868	0	No fix
Nrgn	0	0.868	0	No fix
Penk	0	0.821	0	No fix
Mcpt1	0	0.793	0	No fix
Stfa2l1	0	0.665	0	No fix
Cstdc4	0	0.645	0	No fix
Olr1	1.83E-264	0.765	3.67E-261	No fix
Gm12840	1.38E-234	0.818	2.77E-231	No fix
Ccl6	8.19E-233	0.871	1.64E-229	No fix
Stmn1	7.30E-227	0.918	1.46E-223	No fix
Upp1	4.36E-225	0.762	8.71E-222	No fix
Nlrp3	1.30E-221	0.866	2.59E-218	No fix
Mmp9	3.15E-206	0.773	6.30E-203	No fix
Cd8b1	1.41E-197	0.925	2.82E-194	No fix
Ccl1	4.62E-195	0.784	9.23E-192	No fix
Ccl7	4.57E-189	0.681	9.14E-186	No fix
Slpi	9.59E-189	0.758	1.92E-185	No fix
Ccl3	1.90E-186	0.846	3.81E-183	No fix
Plk3	8.13E-179	0.794	1.63E-175	No fix
Trem1	6.84E-171	0.806	1.37E-167	No fix
Hcar2	3.61E-165	0.781	7.22E-162	No fix
Cma1	1.68E-161	0.796	3.35E-158	No fix
Ccl4	6.11E-143	0.9	1.22E-139	No fix
Lst1	1.81E-138	0.824	3.62E-135	No fix
Ninj1	2.54E-124	0.84	5.09E-121	No fix
Pdcd1	1.01E-101	0.794	2.03E-98	No fix
Hilpda	3.33E-101	0.844	6.65E-98	No fix
G0s2	5.30E-101	0.743	1.06E-97	No fix
Csf1	9.19E-100	0.72	1.84E-96	No fix
Cd24a	1.37E-94	0.774	2.73E-91	No fix
Msrb1	2.01E-92	0.887	4.02E-89	No fix
Marcksl1	3.45E-80	0.737	6.91E-77	No fix
Ccr1	4.68E-68	0.78	9.36E-65	No fix
Isg15	2.25E-61	0.811	4.51E-58	No fix
Cd300lf	7.69E-61	0.705	1.54E-57	No fix
Cwc25	3.11E-49	0.721	6.23E-46	No fix
Ccrl2	5.94E-44	0.845	1.19E-40	No fix
Ier3	2.91E-38	0.819	5.83E-35	No fix
Csf3r	3.34E-36	0.661	6.68E-33	No fix
Acod1	2.34E-29	0.741	4.68E-26	No fix
Ets2	3.42E-25	0.741	6.85E-22	No fix
Ptgs2	1.08E-22	0.705	2.16E-19	No fix
Furin	9.44E-22	0.719	1.89E-18	No fix
S100a8	3.49E-20	0.75	6.97E-17	No fix
Il1r2	1.12E-17	0.75	2.25E-14	No fix
Ifitm1	1.58E-15	0.805	3.16E-12	No fix
Resf1	3.17E-14	0.801	6.34E-11	No fix
Cxcl2	6.98E-12	0.762	1.40E-08	No fix
Egr1	9.74E-11	0.662	1.95E-07	No fix
Cebpb	4.81E-08	0.808	9.63E-05	No fix
Gm100621	7.02E-07	0.538	0.00140427	No fix

gene	p_val	avg_log2FC	p_val_adj	sample
App	1.06E-38	0.276	2.11E-35	DSP fix
Ccdc88a	1.14E-34	0.228	2.29E-31	DSP fix
Prdx1	4.61E-31	0.569	9.23E-28	DSP fix
Psmb9	6.40E-24	0.498	1.28E-20	DSP fix
Ccnd1	9.10E-24	0.215	1.82E-20	DSP fix
Sparc	4.61E-22	0.118	9.22E-19	DSP fix
Pomp	2.01E-21	0.556	4.02E-18	DSP fix
Aif1	1.04E-18	0.254	2.09E-15	DSP fix
Fn1	9.68E-18	0.299	1.94E-14	DSP fix
Tnip3	2.31E-17	0.196	4.61E-14	DSP fix
Ctsc	3.98E-16	0.419	7.95E-13	DSP fix
Nme2	2.00E-15	0.653	4.01E-12	DSP fix
Ifi30	1.66E-11	0.437	3.32E-08	DSP fix
Ms4a6c	3.92E-10	0.29	7.84E-07	DSP fix
H2-DMb1	6.97E-10	0.281	1.39E-06	DSP fix
Cxcl9	3.09E-07	0.331	0.00061754	DSP fix
Pbx1	4.54E-07	0.181	0.00090711	DSP fix
Ccr2	3.09E-06	0.393	0.00617128	DSP fix
Tgfbi	1.18E-05	0.349	0.02359234	DSP fix

gene	p_val	avg_log2FC	p_val_adj	sample
Tpsb2	1.91E-145	0.215	3.83E-142	DSP+DTT
Gm10062	1.39E-54	0.277	2.79E-51	DSP+DTT
Lilrb4a	8.90E-44	0.481	1.78E-40	DSP+DTT
Cd4	2.91E-40	0.242	5.83E-37	DSP+DTT
Tnfrsf4	6.46E-40	0.411	1.29E-36	DSP+DTT
Ikzf2	1.12E-26	0.383	2.25E-23	DSP+DTT
Itgav	9.85E-20	0.275	1.97E-16	DSP+DTT
Rgs1	2.41E-17	0.577	4.83E-14	DSP+DTT
Ctla2a	1.29E-15	0.58	2.57E-12	DSP+DTT
Nrip1	5.01E-14	0.347	1.00E-10	DSP+DTT
Styk1	1.21E-11	0.232	2.41E-08	DSP+DTT
Gm26917	7.60E-11	0.313	1.52E-07	DSP+DTT
Ms4a4b	1.35E-09	0.617	2.71E-06	DSP+DTT
Ighg2b	3.47E-06	0.089	0.00694916	DSP+DTT
Kit	1.68E-05	0.225	0.03365845	DSP+DTT

DEGs T cell cluster

gene	p_val	avg_log2FC	p_val_adj	sample
Ccl3	0	0.874	0	No fix
Mcpt4	0	0.723	0	No fix
Ccl1	0	0.835	0	No fix
Hcar2	0	0.851	0	No fix
Gm12840	0	0.863	0	No fix
Penk	0	0.76	0	No fix
Mmp9	0	0.76	0	No fix
Retnlg	0	0.683	0	No fix
Hist1h1b	0	0.9	0	No fix
Gzmf	0	0.752	0	No fix
Slpi	0	0.757	0	No fix
Nrgn	0	0.848	0	No fix
Plk3	0	0.814	0	No fix
Il1a	0	0.691	0	No fix
Slfn1	0	0.849	0	No fix
Gm20186	0	0.75	0	No fix
Cyp11a1	1.86E-289	0.649	3.72E-286	No fix
Mcpt1	6.24E-287	0.647	1.25E-283	No fix
Cwc25	7.54E-277	0.835	1.51E-273	No fix
Ccl6	6.55E-261	0.8	1.31E-257	No fix
Pilra	1.23E-255	0.679	2.46E-252	No fix
Ccl4	1.11E-243	0.879	2.23E-240	No fix
Trem1	1.03E-236	0.748	2.07E-233	No fix
Clec4n	2.23E-225	0.725	4.47E-222	No fix
Rgcc	7.26E-215	0.796	1.45E-211	No fix
Clec4d	5.39E-202	0.766	1.08E-198	No fix
G0s2	4.22E-173	0.731	8.45E-170	No fix
Cxcr2	3.69E-165	0.632	7.37E-162	No fix
Stmn1	1.04E-159	0.806	2.08E-156	No fix
Cd8a	3.21E-154	0.827	6.42E-151	No fix
Csf3r	5.22E-145	0.696	1.04E-141	No fix
Marcksl1	1.18E-140	0.743	2.36E-137	No fix
Pglyrp1	5.10E-140	0.91	1.02E-136	No fix
Cma1	1.45E-133	0.699	2.90E-130	No fix
Hdc	4.00E-132	0.769	8.00E-129	No fix
Clec5a	2.15E-121	0.654	4.31E-118	No fix
Ifitm1	2.14E-120	0.889	4.28E-117	No fix
Ccl7	1.71E-119	0.612	3.41E-116	No fix
Hilpda	3.65E-113	0.819	7.31E-110	No fix
Slc7a11	2.88E-105	0.666	5.77E-102	No fix
Ier3	1.83E-100	0.83	3.65E-97	No fix
Isg15	8.53E-99	0.788	1.71E-95	No fix
Csf1	1.06E-77	0.67	2.12E-74	No fix
Rsad2	8.10E-73	0.643	1.62E-69	No fix
Ltb	4.04E-42	0.925	8.09E-39	No fix
Ccrl2	8.35E-42	0.79	1.67E-38	No fix
Clec4e	9.54E-41	0.71	1.91E-37	No fix
Bcl2a1b	7.29E-37	0.925	1.46E-33	No fix
Cd9	1.18E-32	0.733	2.36E-29	No fix
Tnfaip2	3.54E-27	0.733	7.09E-24	No fix
Cd3g	3.71E-27	0.891	7.42E-24	No fix
Tpsb2	4.75E-24	0.614	9.49E-21	No fix
Il1r2	2.85E-23	0.728	5.70E-20	No fix
Mcpt2	2.27E-13	0.709	4.55E-10	No fix
Ptgs2	3.92E-12	0.553	7.84E-09	No fix
Acod1	1.03E-11	0.667	2.05E-08	No fix
Cebpb	5.63E-11	0.802	1.13E-07	No fix
Gadd45b	2.72E-10	0.761	5.44E-07	No fix
Cxcl2	8.05E-09	0.725	1.61E-05	No fix
Cxcl10	1.85E-08	0.642	3.71E-05	No fix
AY036118	2.15E-08	0.894	4.29E-05	No fix
S100a9	3.78E-07	0.775	0.00075667	No fix
Egr1	4.40E-07	0.574	0.00088015	No fix

gene	p_val	avg_log2FC	p_val_adj	sample
Plekho1	2.58E-239	0.193	5.16E-236	DSP fix
Ifi211	8.16E-231	0.165	1.63E-227	DSP fix
Tnip3	3.73E-226	0.138	7.46E-223	DSP fix
Ccl17	8.65E-220	0.133	1.73E-216	DSP fix
Pkib	1.08E-185	0.165	2.17E-182	DSP fix
Cbfa2t3	9.29E-142	0.147	1.86E-138	DSP fix
Ckb	1.68E-129	0.239	3.36E-126	DSP fix
Fcgr2b	1.35E-117	0.154	2.70E-114	DSP fix
Sdc4	5.43E-108	0.151	1.09E-104	DSP fix
Ifi205	1.30E-107	0.132	2.60E-104	DSP fix
Arg1	1.21E-103	0.116	2.43E-100	DSP fix
Gng10	8.99E-102	0.361	1.80E-98	DSP fix
Ms4a6d	3.95E-80	0.188	7.90E-77	DSP fix
Pld4	4.59E-80	0.241	9.19E-77	DSP fix
Nupr1	1.27E-77	0.153	2.55E-74	DSP fix
Csf1r	2.84E-76	0.255	5.69E-73	DSP fix
Ms4a6c	6.34E-73	0.234	1.27E-69	DSP fix
H2-DMa	8.90E-69	0.325	1.78E-65	DSP fix
H2-DMb1	1.07E-66	0.213	2.13E-63	DSP fix
Cd86	1.77E-66	0.214	3.54E-63	DSP fix
Klf4	1.74E-65	0.252	3.48E-62	DSP fix
Fn1	3.69E-63	0.223	7.39E-60	DSP fix
Ifi207	9.98E-61	0.172	2.00E-57	DSP fix
Anxa1	1.13E-59	0.241	2.26E-56	DSP fix
Napsa	1.40E-59	0.255	2.80E-56	DSP fix
Etv3	2.98E-57	0.201	5.96E-54	DSP fix
Wfdc17	3.06E-54	0.19	6.11E-51	DSP fix
Gpr141	3.47E-49	0.157	6.94E-46	DSP fix
Atox1	2.81E-45	0.529	5.61E-42	DSP fix
Gpx1	3.88E-45	0.495	7.76E-42	DSP fix
Gatm	1.57E-44	0.115	3.14E-41	DSP fix
Mmp14	2.05E-44	0.092	4.10E-41	DSP fix
Ptms	1.54E-42	0.427	3.09E-39	DSP fix
Msr1	1.22E-41	0.122	2.44E-38	DSP fix
Bach1	8.78E-40	0.155	1.76E-36	DSP fix
Ctss	1.55E-39	0.546	3.09E-36	DSP fix
Ifi30	2.05E-38	0.394	4.09E-35	DSP fix
Gm2a	3.20E-35	0.385	6.39E-32	DSP fix
Prdx1	1.11E-29	0.552	2.22E-26	DSP fix
Irf8	5.36E-29	0.401	1.07E-25	DSP fix
Aif1	1.63E-28	0.226	3.27E-25	DSP fix
Alox5ap	8.20E-28	0.35	1.64E-24	DSP fix
Mt1	2.82E-27	0.225	5.63E-24	DSP fix
Pomp	2.89E-27	0.551	5.77E-24	DSP fix
Ms4a4c	2.76E-25	0.272	5.52E-22	DSP fix
Ccr2	7.35E-25	0.355	1.47E-21	DSP fix
Hsp90b1	2.71E-24	0.593	5.42E-21	DSP fix
Cxcl16	1.47E-23	0.237	2.95E-20	DSP fix
Tns3	1.05E-22	0.12	2.10E-19	DSP fix
C1qb	4.41E-22	0.18	8.81E-19	DSP fix
Cybb	5.69E-21	0.236	1.14E-17	DSP fix
Ctsc	2.00E-19	0.362	4.01E-16	DSP fix
Nme2	2.97E-18	0.654	5.94E-15	DSP fix
App	3.60E-17	0.196	7.20E-14	DSP fix
Ctsz	4.67E-17	0.378	9.33E-14	DSP fix
Lgals3	9.80E-17	0.518	1.96E-13	DSP fix
Ctsh	1.64E-15	0.259	3.27E-12	DSP fix
C1qa	3.67E-15	0.144	7.33E-12	DSP fix
Mafb	1.17E-13	0.176	2.35E-10	DSP fix
Psap	1.27E-13	0.546	2.53E-10	DSP fix
Cxcl9	3.40E-13	0.243	6.79E-10	DSP fix
Calm1	4.97E-13	0.825	9.94E-10	DSP fix
Cst3	5.45E-12	0.756	1.09E-08	DSP fix
Crip1	4.98E-11	0.74	9.95E-08	DSP fix
Calr	1.21E-10	0.621	2.42E-07	DSP fix
Clic4	1.48E-09	0.267	2.97E-06	DSP fix
Ifitm3	1.26E-05	0.522	0.0252623	DSP fix
Plbd1	1.58E-05	0.276	0.03163834	DSP fix
H2-Aa	1.96E-05	0.48	0.03917465	DSP fix

gene	p_val	avg_log2FC	p_val_adj	sample
Ighg2c	2.20E-208	0.209	4.40E-205	DSP+DTT
Pkib1	2.45E-185	0.201	4.90E-182	DSP+DTT
Nedd4	3.46E-185	0.184	6.91E-182	DSP+DTT
Fn11	6.51E-143	0.191	1.30E-139	DSP+DTT
Gm36723	6.43E-86	0.241	1.29E-82	DSP+DTT
Ccl22	3.09E-84	0.173	6.18E-81	DSP+DTT
Apoe	1.40E-72	0.377	2.79E-69	DSP+DTT
Il7r	1.18E-57	0.343	2.35E-54	DSP+DTT
Gzma	4.22E-54	0.235	8.45E-51	DSP+DTT
Neb	4.76E-33	0.141	9.52E-30	DSP+DTT
Ighg2b	5.09E-30	0.051	1.02E-26	DSP+DTT
Gm26917	5.07E-23	0.294	1.01E-19	DSP+DTT
Ifitm31	1.95E-21	0.518	3.91E-18	DSP+DTT
Txk	2.26E-18	0.312	4.53E-15	DSP+DTT
Fscn1	1.26E-12	0.101	2.51E-09	DSP+DTT
Serpinb9	6.79E-11	0.443	1.36E-07	DSP+DTT
Lrba	1.14E-08	0.208	2.29E-05	DSP+DTT
H2-Ab11	4.75E-06	0.569	0.00949117	DSP+DTT

DEGs DC cluster

gene	p_val	avg_log2FC	p_val_adj	sample
Pdgfb	0	0.53224382	0	No fix
Iglc2	0	0.50867264	0	No fix
Cd7	0	0.44043037	0	No fix
Mcpt1	0	0.41090003	0	No fix
Ighg2c	0	0.3598733	0	No fix
Ccl3	0	0.3145301	0	No fix
Trem2	0	0.30901228	0	No fix
Cd68	0	0.30663993	0	No fix
Chil3	0	0.2870499	0	No fix
Mcpt4	0	0.28598216	0	No fix
Col1a2	0	0.2768762	0	No fix
Col1a1	0	0.27592026	0	No fix
Ccl4	0	0.26435387	0	No fix
Ly6i	0	0.26260357	0	No fix
Pilra	0	0.25683765	0	No fix
Cyp11a1	0	0.25154645	0	No fix
Ccl9	1.16E-264	0.3449169	2.32E-261	No fix
Ccl6	2.09E-258	0.36205256	4.17E-255	No fix
C1qc	6.31E-213	0.31123557	1.26E-209	No fix
Irf7	1.56E-182	0.29176543	3.12E-179	No fix
Csf1r	5.22E-160	0.2789305	1.04E-156	No fix
Igsf6	7.11E-136	0.31262493	1.42E-132	No fix
Cd74	8.31E-132	0.41487366	1.66E-128	No fix
Fcgr3	1.94E-129	0.30471736	3.88E-126	No fix
C1qb	5.61E-97	0.28437256	1.12E-93	No fix
H2-Ab1	1.23E-91	0.29325651	2.46E-88	No fix
H2-Aa	5.28E-91	0.27289523	1.06E-87	No fix
Tpsb2	2.13E-39	0.3694829	4.25E-36	No fix
Gm100621	8.96E-23	0.31286575	1.79E-19	No fix

gene	p_val	avg_log2FC	p_val_adj	sample
Bst2	3.95E-145	0.28209873	7.90E-142	DSP fix
Gpx1	8.21E-134	0.33762723	1.64E-130	DSP fix
Pfn1	1.99E-125	0.42457293	3.99E-122	DSP fix
Rpl10	3.86E-99	0.32961645	7.71E-96	DSP fix
Ms4a6c	3.85E-68	0.34000587	7.71E-65	DSP fix
Bcl2a1b	1.63E-54	0.32007875	3.25E-51	DSP fix
Iigp1	8.87E-49	0.28182372	1.77E-45	DSP fix
Prdx5	1.27E-48	0.33277775	2.55E-45	DSP fix
Txn1	1.21E-46	0.29241143	2.42E-43	DSP fix
Calm1	6.08E-42	0.26454293	1.22E-38	DSP fix
Ccl7	1.65E-24	0.42457957	3.31E-21	DSP fix
Bcl2a1d	5.79E-24	0.27578033	1.16E-20	DSP fix
Aif1	1.03E-09	0.27492603	2.07E-06	DSP fix

gene	p_val	avg_log2FC	p_val_adj	sample
Cemip2	3.69E-156	0.28230455	7.38E-153	DSP+DTT
Gm10062	4.94E-103	0.40908598	9.89E-100	DSP+DTT
St8sia4	1.28E-41	0.25630963	2.55E-38	DSP+DTT
Odc1	6.32E-39	0.26462361	1.26E-35	DSP+DTT
Trp53inp1	1.61E-26	0.29209367	3.21E-23	DSP+DTT
Ttn	4.01E-23	0.38668286	8.03E-20	DSP+DTT
Nkg7	2.79E-14	0.31152671	5.59E-11	DSP+DTT
Gm26917	2.52E-13	1.01276511	5.04E-10	DSP+DTT
Tmsb10	2.53E-11	0.45245152	5.05E-08	DSP+DTT
Rora	4.41E-10	0.40752165	8.82E-07	DSP+DTT
Vim	6.38E-10	0.34748881	1.28E-06	DSP+DTT
Fth1	2.56E-07	0.2570774	0.00051104	DSP+DTT
Zfp36	2.83E-07	0.25266258	0.00056627	DSP+DTT
Vmn1r13	2.54E-06	0.27800022	0.0050812	DSP+DTT
Igkc	7.05E-06	0.33118425	0.01410551	DSP+DTT
Rps20	9.62E-06	0.33946382	0.01923001	DSP+DTT
Hsp90ab1	2.28E-05	0.29923002	0.04564179	DSP+DTT

DEGs NK cell cluster

gene	p_val	avg_log2FC	p_val_adj	sample
Iglc2	0	0.30949785	0	No fix
Stfa2l1	0	0.2763619	0	No fix
Serpinb2	4.25E-247	0.31782128	8.51E-244	No fix
Il13	4.69E-227	0.33688678	9.37E-224	No fix
Il6	3.85E-220	0.25711661	7.69E-217	No fix
Mcpt8	6.05E-166	0.26007499	1.21E-162	No fix
Mmp9	5.66E-161	0.32312752	1.13E-157	No fix
Mcpt1	7.47E-154	0.92970133	1.49E-150	No fix
Hist1h1b	7.91E-134	0.55524767	1.58E-130	No fix
Osm	6.88E-118	0.27716851	1.38E-114	No fix
Clec4n	1.66E-110	0.38204265	3.32E-107	No fix
Csf1	2.24E-104	0.43200265	4.48E-101	No fix
C1qc	1.76E-102	0.25739331	3.53E-99	No fix
Acp5	7.01E-99	0.26872375	1.40E-95	No fix
Nrgn	5.51E-96	0.33948998	1.10E-92	No fix
Ccl3	9.35E-91	1.04908374	1.87E-87	No fix
Med21	2.75E-89	0.38053358	5.51E-86	No fix
1110008P14	7.44E-86	0.29725484	1.49E-82	No fix
Mcpt4	1.66E-85	0.30919552	3.33E-82	No fix
Gm20186	3.83E-83	0.40764974	7.66E-80	No fix
Rgcc	3.89E-81	0.2831684	7.78E-78	No fix
Slpi	7.90E-81	0.28026075	1.58E-77	No fix
Top2a	1.09E-77	0.26868885	2.18E-74	No fix
Olr1	8.61E-75	0.31399304	1.72E-71	No fix
Cd8a	3.23E-72	0.31000793	6.45E-69	No fix
Dnajb1	5.21E-71	0.31123327	1.04E-67	No fix
Plk3	6.03E-67	0.25328103	1.21E-63	No fix
Ccl4	2.10E-66	1.1356198	4.19E-63	No fix
Csf3r	3.68E-65	0.29589448	7.37E-62	No fix
Cd8b1	7.54E-62	0.29506514	1.51E-58	No fix
Serpina3g	3.51E-60	0.27007557	7.03E-57	No fix
Gm12840	1.35E-59	0.42863891	2.71E-56	No fix
Cxcl10	2.64E-59	0.38872001	5.28E-56	No fix
Ccl6	2.21E-58	0.50817401	4.42E-55	No fix
Ndufs7	6.35E-56	0.31241708	1.27E-52	No fix
Hilpda	2.06E-55	0.33915004	4.12E-52	No fix
Zbp1	5.69E-55	0.26999652	1.14E-51	No fix
Ier3	2.89E-54	0.46767597	5.78E-51	No fix
Clec4e	5.56E-53	0.26921725	1.11E-49	No fix
Hspa1a	6.14E-53	0.35473967	1.23E-49	No fix
Pdgfb	8.92E-51	0.27030967	1.78E-47	No fix
Clec4d	1.18E-50	0.30029193	2.37E-47	No fix
Ddt	7.02E-49	0.32054769	1.40E-45	No fix
Isg15	1.54E-47	0.37910757	3.07E-44	No fix
Ncf1	2.15E-47	0.25215433	4.31E-44	No fix
Ets2	3.45E-41	0.27503134	6.91E-38	No fix
Stmn1	5.59E-41	0.41902202	1.12E-37	No fix
Acod1	1.08E-40	0.45113134	2.16E-37	No fix
Ccr1	1.10E-40	0.29730218	2.20E-37	No fix
Hist1h1e	2.68E-40	0.32485023	5.36E-37	No fix
Ccrl2	4.00E-40	0.33569789	8.01E-37	No fix
Hcar2	1.10E-34	0.45934976	2.20E-31	No fix
Cxcl2	1.31E-31	0.40679951	2.63E-28	No fix
Rsad2	1.81E-31	0.41439333	3.61E-28	No fix
Cdkn1a	1.08E-29	0.25781312	2.16E-26	No fix
Klf2	2.46E-27	0.33614715	4.91E-24	No fix
Gzmb	3.31E-27	0.52158663	6.63E-24	No fix
Ifitm1	4.25E-27	0.53110643	8.50E-24	No fix
Bcl2a1b	5.27E-26	0.27794134	1.05E-22	No fix
Tpsb2	6.03E-26	0.2943619	1.21E-22	No fix
Nfkbiz	4.50E-23	0.26719725	9.00E-20	No fix
Gzma	5.09E-21	0.40646338	1.02E-17	No fix
G0s2	8.95E-20	0.56077929	1.79E-16	No fix
Ptgs2	1.44E-16	0.31421507	2.89E-13	No fix
Cd68	1.50E-16	0.30924208	2.99E-13	No fix
Pde6a	2.99E-16	0.28670969	5.98E-13	No fix
Cd7	5.08E-14	0.43239049	1.02E-10	No fix
Trbc1	1.61E-10	0.25620262	3.22E-07	No fix
Cks2	3.42E-10	0.35403291	6.85E-07	No fix
Hdc	2.09E-06	0.5394879	0.00418772	No fix
Cxcr4	2.17E-06	0.30702493	0.00434296	No fix
Resf1	5.81E-06	0.32565389	0.01161914	No fix

gene	p_val	avg_log2FC	p_val_adj	sample
Pid1	2.74E-73	0.26494698	5.49E-70	DSP fix
Tbc1d4	7.02E-57	0.50677003	1.40E-53	DSP fix
Cma1	4.46E-14	0.29213415	8.92E-11	DSP fix
Fn1	1.19E-09	0.52086682	2.38E-06	DSP fix

gene	p_val	avg_log2FC	p_val_adj	sample
Fscn1	4.14E-44	0.26875811	8.29E-41	DSP+DTT
Plcxd2	1.80E-34	0.26677497	3.60E-31	DSP+DTT
Iqcb1	1.44E-24	0.25676874	2.89E-21	DSP+DTT
Ighm	7.27E-13	0.3994905	1.45E-09	DSP+DTT
Mc1r	1.40E-12	0.37196492	2.80E-09	DSP+DTT
Ctss	3.64E-08	0.2596026	7.28E-05	DSP+DTT
Clic4	7.95E-08	0.27451817	0.00015903	DSP+DTT
Samhd1	8.38E-08	0.30196521	0.00016764	DSP+DTT
Lyz2	2.03E-07	0.48482131	0.0004057	DSP+DTT
